# Can antibiotics for enteritis or for urinary tract infection disrupt the urinary microbiota in rats?

**DOI:** 10.3389/fcimb.2023.1169909

**Published:** 2023-06-28

**Authors:** Fengping Liu, Lei Hu, Jiayi Sheng, Yifan Sun, Qiang Xia, Yifan Tang, Peng Jiang, Shichao Wei, Jialin Hu, Hao Lin, Zhenyi Xu, Wei Guo, Yifeng Gu, Ninghan Feng

**Affiliations:** ^1^ Wuxi School of Medicine, Jiangnan University, Wuxi, Jiangsu, China; ^2^ Department of Urology, Affiliated Wuxi No. 2 Hospital, Nanjing Medical University, Wuxi, Jiangsu, China; ^3^ Jiangnan University Medical Center, Wuxi, Jiangsu, China; ^4^ Department of Urology, Wuxi 9 People’s Hospital Affiliated to Soochow University, Wuxi, Jiangsu, China; ^5^ Collaborative Innovation Center for Diagnosis and Treatment of Infectious Diseases, State Key Laboratory for Diagnosis and Treatment of Infectious Diseases, School of Medicine, The First Affiliated Hospital, Zhejiang University, Hangzhou, Zhejiang, China

**Keywords:** antibiotics, enteritis, gavage, microbiota, urinary catheterization, urinary tract infection

## Abstract

**Background:**

To establish antibiotic preregimes and administration routes for studies on urinary microbiota.

**Methods and materials:**

Antibiotics for enteritis (Abx-enteritis) and UTIs (Abx-UTI) were administered via gavage and/or urinary catheterisation (UC) for 1 and/or 2 weeks. The effects of these Abx on the urinary microbiota of rats were examined via 16S rRNA sequencing and urine culture, including anaerobic and aerobic culture. Additionally, the safety of the Abx was examined.

**Results:**

Abx-enteritis/Abx-UTI (0.5 g/L and 1 g/L) administered via gavage did not alter the microbial community and bacterial diversity in the urine of rats (FDR > 0.05); however, Abx-UTI (1 g/L) administered via UC for 1 and 2 weeks altered the urinary microbial community (FDR < 0.05). Rats administered Abx-UTI (1 g/L) via UC for 1 week demonstrated a distinct urinary microbiota in culture. Abx-enteritis/Abx-UTI administered via gavage disrupted the microbial community and reduced bacterial diversity in the faeces of rats (FDR < 0.05), and Abx-UTI administered via UC for 2 weeks (FDR < 0.05) altered the fecal microbiota. Abx-UTI (1 g/L) administered via UC did not alter safety considerations. In addition, we noticed that UC did not induce infections and injuries to the bladder and kidney tissues.

**Conclusions:**

Administration of Abx-UTI via UC for 1 week can be considered a pre-treatment option while investigating the urinary microbiota.

## 1 Introduction

Perturbations in the composition and function of the human microbiota have been associated with diseases. To elucidate the role and underlying mechanisms of microbiota in diseases, studies are now focusing on the causal relationship between microbiota and diseases via experimental manipulation of the microbiota. Microbiota transplantation (MT) is one of the most commonly used approaches for investigating the causal relationship between microbiota and diseases in animal models (Fischbach, 2018).

For optimizing MT for diseases, germ free animals or animals with antibiotics pre-treatment can enhance the efficacy of MT (Turnbaugh et al., 2009; Manichanh et al., 2010; Wos-Oxley et al., 2012; Hintze et al., 2014). However, germ-free mice/rats are expensive, and specialized genetic strains require specialized facilities and expertise, limiting their widespread use (Cho and Blaser, 2012). Thus, antibiotic-treated mice/rats are alternative animal models that can be used for investigating the role of microbiota in preclinical studies (Lundberg et al., 2016).

Antibiotics for pre-treatment are often selected by targeted manipulation of body niches. For example, antibiotics used to treat enteritis (Abx-enteritis) (Rakoff-Nahoum et al., 2004; Kathania et al., 2020), whereas antibiotics used to treat vaginitis are used for vaginal MT (Chen et al., 2021).

Over the past decade, studies have demonstrated that the human bladder possesses a microbial community (Wolfe et al., 2012; Hilt et al., 2014; Pearce et al., 2015; Liu et al., 2022). However, to date, the urinary microbiota of humans and animals has been analysed only based on observation. Thus, the cause-and-effect relationship between the urinary microbiota and diseases remains unclear.

Because broad-spectrum Abx-enteritis can disrupt the gut microbiota, they can be used to understand the role of the gut microbiota in pathological conditions (Rakoff-Nahoum et al., 2004; Desbonnet et al., 2015); however, whether Abx-enteritis can also disrupt the urinary microbiota remains unknown. Additionally, whether antibiotics used to treat UTIs (Abx-UTI) are more suitable for disrupting the urinary microbiota remains unknown. It is necessary to establish a safe method to disrupt the urinary microbiota in animals, which can facilitate the study of the impacts of urinary microbiota on health.

## 2 Materials and methods

### 2.1 Animals

Female Sprague-Dawley rats (age, 8 weeks; weight, 280 g) were used. Female rats were used because they can be easily administered drugs via urinary catheterisation (UC), and approximately 1 mL of urine, which is required to yield sufficient bacterial DNA for sequencing and culture, can be collected (Bordelon et al., 2013; Pearce et al., 2015).

### 2.2 Antibiotic treatment

Abx-enteritis or Abx-UTI dissolved in drinking water were administered via gavage, and Abx-UTI in normal saline (NS) were administered via UC. Abx-enteritis, including ampicillin, vancomycin, neomycin and metronidazole, were selected (Rakoff-Nahoum et al., 2004; Baldridge et al., 2015). Abx-UTI, including fosfomycin, nitrofurantoin, gentamicin, cefotaxime, and metronidazole (Naber et al., 2001).

The rats were randomly divided into 11 groups. 1) Abx-enteritis-0.5g-G-2W: Abx-enteritis at a dose of 0.5 g/L were dissolved in water and administered to acclimatised rats once every 7 days via gavage; the rats were dissected at the end of week 2. 2) Abx-enteritis-1g-G-2W: Abx-enteritis at a dose of 1 g/L were dissolved, and the subsequent protocol was the same as that for the Abx-enteritis-0.5g-G-2W group. 3) Abx-UTI-0.5g-G-2W: Abx-UTI at a dose of 0.5 g/L were dissolved, and the subsequent protocol was the same as that for the Abx-enteritis-0.5g-G-2W group. 4) Abx-UTI-1g-G-2W: Abx-UTI at a dose of 1 g/L were dissolved, and the subsequent protocol was the same as that for the Abx-enteritis-0.5g-G-2W group. 5) Abx-UTI-0.5g-UC-2W: NS containing 0.5-g/L Abx-UTI was administered via UC. UC was performed once every 7 days, and the rats were dissected at the end of week 2. 6) Abx-UTI-1g-UC-2W: NS containing 1-g/L Abx-UTI was administered via UC, and the subsequent protocol was the same as that for the Abx-UTI-0.5g-UC-2W group. 7) Abx-UTI-0.5g-UC-1W: The protocol was the same as that for the Abx-UTI-0.5g-UC-2W group; however, the experiment lasted only 1 week. 8) Abx-UTI-1g-UC-1W: NS containing 1-g/L Abx-UTI was administered via UC, and the remaining protocol was the same as that for the Abx-UTI-0.5g-UC-1W group. 9) Control: Rats were not administered any antibiotics. 10) NS-UC-2W (sham group): NS was administered via UC once every 7 days, and the rats were dissected at the end of week 2. 11) NS-UC-1W (sham group): NS was administered via UC after acclimatization, and the rats were dissected at the end of week 2. All groups with antibiotic treatment contained 9 rats, whereas the control and the two sham groups contained 6 rats.

For the UC groups, the rats were anaesthetised via intraperitoneal injection of 1% pentobarbitone. Under anaesthesia, the rats were positioned in dorsal recumbence. A 26-gauge polyurethane IV catheter was used for UC. Rats under UC were placed in a biosafety cabinet, and 2% iodine tincture was used to disinfect the abdominal, genital and perineal areas twice.

### 2.3 Detection of urinary and faecal microbiota

The rats were dissected under anaesthesia, and fresh urine and faeces were collected. Methods of urine collection and storage, bacterial DNA isolation and bioinformatic analysis, urine culture and bacterial identification are described in **File S1**.

### 2.4 Assessment of the safety of antibiotics

#### 2.4.1 Processing and haematoxylin–eosin staining of rat tissues

The structures of the bladder and kidney were analyzed to examine the safety profile of Abx-enteritis and Abx-UTI. The kidney and bladder tissues of rats were collected and soaked in 4% paraformaldehyde for external fixation. Subsequently, the tissues were dehydrated, permeabilized, embedded and cut into 2-μm-thick sections. The slices were collected at intervals of six, and three slices were placed on each glass slide for staining. A haematoxylin–eosin (H&E) staining kit (Cat. G1120, Solarbio, Beijing Solarbio Science & Technology, Beijing, China) was used for histopathological examination. The experiment was performed following the standard operating procedures.

#### 2.4.2 Assessment of kidney function, body weight, food and water consumption and faeces and urine output

Kidney function, body weight gain, intake of food and water and output of urine and faeces were also evaluated to examine the safety profile of Abx-enteritis and Abx-UTI. Blood samples were collected from the inferior vein after the rats were dissected. The samples were centrifuged at 3000 ×g to separate the serum. Kidney function was examined by evaluating the levels of serum creatinine, uric acid and blood urea using an automated biochemical analyser (Olympus, AU5421, Tokyo, Japan).

The body weight of the rats was measured twice: after acclimation and before the day of dissection. The intake of food and water was assessed before the day of dissection. Faeces and urine samples were collected from rats housed in metabolic cages 24 h before dissection. During sample collection, the rats were allowed free access to food and water.

### 2.5 Statistics analysis

Sequence data analysis was performed using QIIME and R package (V3.2.0). Beta diversity analysis was performed to investigate the structural variation of fungal communities across samples using Bray-Curtis metrics and visualized via principal coordinate analysis (PCoA) based on permutational multivariate analysis of variance [PERMANOVA] calculated by ‘adonis’ function. ASV-level alpha richness and diversity indices, including Chao 1 and Shannon, were calculated using the ASV table.

Wilcoxon rank-sum test was used on the comparison of bacterial diversity and taxon between groups. ANOVA were used on the variables of kidney function estimators among groups. The *P*-value was adjusted for multiple comparisons using the Benjamini–Hochberg (BH) false discovery rate (FDR). The significance threshold was set at an FDR-corrected value <0.05.

## 3 Results

### 3.1 Effects of antibiotics on urinary microbiota

#### 3.1.1 Abx-enteritis and Abx-UTI administered via gavage did not alter urinary microbiota

##### 3.1.1.1 Administration of Abx-enteritis via gavage

Abx-enteritis at the doses of 0.5 g/L and 1 g/L were administered via gavage for 2 weeks (Abx-enteritis-0.5g-G-2W and Abx-enteritis-1g-G-2W groups, respectively), and their effects on the urinary microbiota were examined.

**Figure 1:**
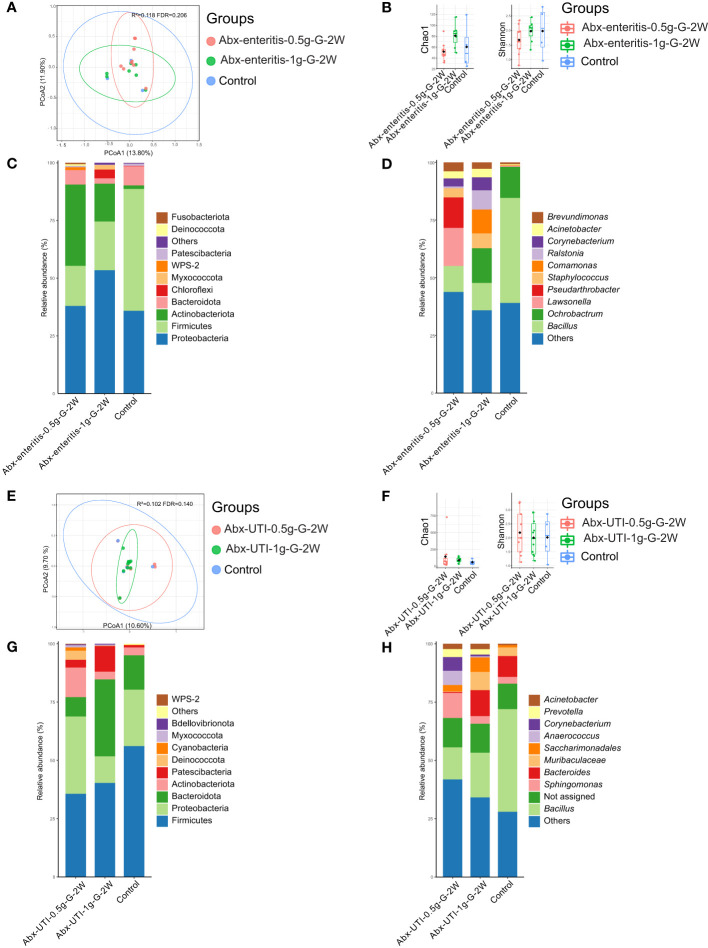
Urinary microbiota alterations in rats administered antibiotics *via* gavage. **(A)** PCoA based on Bray-Curtis distances ASV level showed comparisons of microbial community among groups of Abx-enteritis-0.5g-G-2W, Abx-enteritis-1g-G-2W and controls. **(B)** Bacterial richness and diversity measured by Chao 1 and Shannon index were calculated at ASV level, and compared among groups of Abx-enteritis-0.5g-G-2W, Abx-enteritis-1g-G-2W and controls. **(C)** Microbial profile at the phylum level of groups of Abx-enteritis-0.5g-G-2W, Abx-enteritis-1g-G-2W and controls. Only the top 5 most abundant phyla are shown. **(D)** Microbial profile at the genus level of the groups of Abx-enteritis-0.5g-G-2W, Abx-enteritis-1g-G-2W and controls. Only the top 10 most abundant genera are shown. **(E)** PCoA based on Bray-Curtis distances ASV level showed comparisons of microbial community among groups of Abx-UTI-0.5g-G-2W, Abx-UTI-1g-G-2W and controls. **(F)** Bacterial richness and diversity measured by Chao 1 and Shannon index were calculated at ASV level, and compared among groups of Abx-UTI-0.5g-G-2W, Abx-UTI-1g-G-2W and controls. **(G)** Microbial profile at the phylum level of groups Abx-UTI-0.5g-G-2W, Abx-UTI-1g-G-2W and controls. Only the top 5 most abundant phyla are shown. **(H)** Microbial profile at the genus level of groups of Abx-UTI-0.5g-G-2W, Abx-UTI-1g-G-2W and controls. Only the top 10 most abundant genera are shown.

The composition of the bacterial community in urine was not significantly different between the Abx-enteritis and control groups (R2 = 0.118, FDR > 0.05) (**Figure 1A**). Additionally, bacterial richness (Chao 1 index) and bacterial diversity (Shannon index) were not significantly different between the two groups (FDR > 0.05) (**Figure 1B**). The dominant bacterial phyla were Firmicutes (52.86%) and Proteobacteria (35.82%) in the control group (**Figure 1C**). Firmicutes and Proteobacteria were also ranked among the top three most abundant bacterial phyla in the Abx-enteritis-0.5g-G-2W and Abx-enteritis-1g-G-2W groups. No significant difference was observed in the abundance of bacterial phyla between the Abx-enteritis and control groups (*FDR > 0.05)*. Bacillus (45.50%) was the dominant genus in the control group (**Figure 1D**), whereas it was one of the most abundant bacterial genera in the two Abx-enteritis groups, representing 5.80% and 7.73% of the total bacterial genera in the Abx-enteritis-0.5g-G-2W and Abx-enteritis-1g-G-2W groups, respectively. However, *no significant difference was observed in the abundance of bacterial genera* accounting for >1% of the total bacterial composition *between the* Abx-enteritis *and control groups* (*FDR > 0.05).*

##### 3.1.1.2 Administration of Abx-UTI via gavage

On the one hand, no significant difference was found in the composition of the bacterial community (R^2 = ^0.102, FDR = 0.140) (**Figure 1E**) and bacterial diversity between the Abx-UTI (Abx-UTI-0.5g-G-2W and Abx-UTI-1g-G-2W) and control groups (FDR > 0.05) (**Figure 1F**). On the other hand, the abundance of Firmicutes was higher in the control group than in the two Abx-UTI groups (**Figure 1G**); however, the difference was not significant (FDR > 0.05). In addition, the enrichment of Proteobacteria in Abx-UTI-0.5g-G-2W group and the depletion of Proteobacteria in the Abx-UTI-1g-G-2W group were not significantly different compared with the control group (FDR > 0.05). Sphingomonas (11.94%) was the most abundant bacterial genus in the Abx-UTI-0.5g-G-2W group, and Bacillus was the most abundant genus in the Abx-UTI-1g-G-2W and control groups (**Figure 1H**). No significant differences were found between the Abx-UTI and control groups at the genus level (FDR > 0.05).

#### 3.1.2 Abx-UTI administered via UC altered urinary microbiota

##### 3.1.2.1 Administration of Abx-UTI via UC for 2 weeks

Administration of Abx-enteritis and Abx-UTI in drinking water did not alter the microbial community in the urine of rats; therefore, Abx-UTI were administered via UC once a week for either 1 week or 2 weeks.

**Figure 2:**
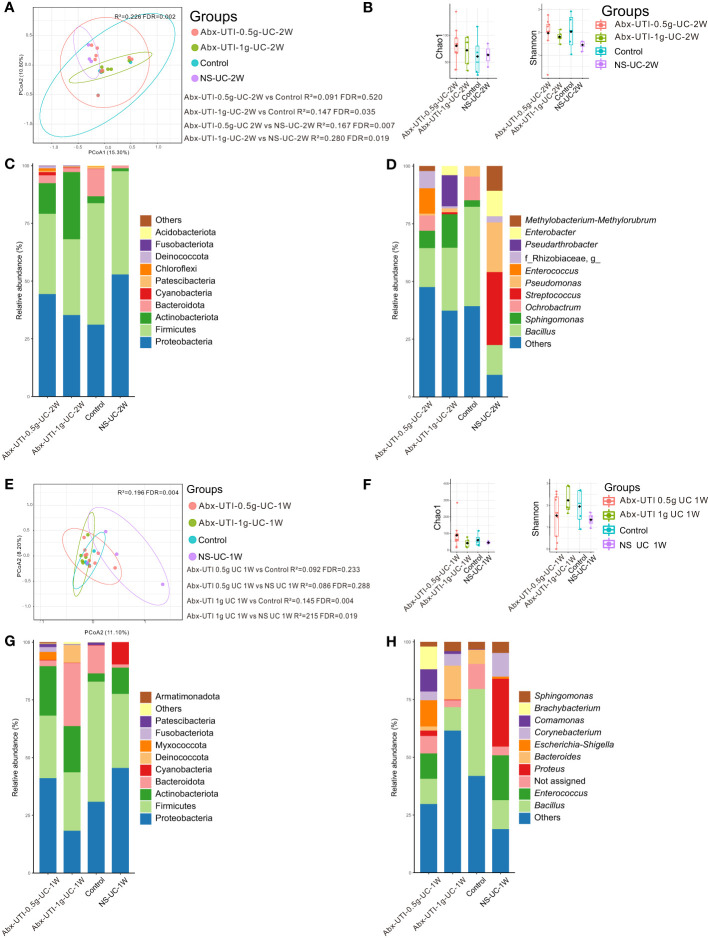
Urinary microbiota alterations in rats administered antibiotics for UTI via UC. **(A)** PCoA based on Bray-Curtis distances ASV level showed comparisons of microbial community among groups of Abx-UTI-0.5g-UC-2W, Abx-UTI-1g-UC-2W, Controls, and NS-UC-2W. **(B)** Bacterial richness and diversity measured by Chao 1 and Shannon index were calculated at ASV level, and compared among groups of Abx-UTI-0.5g-UC-2W, Abx-UTI-1g-UC-2W, Controls, and NS-UC-2W. **(C)** Microbial profile at the phylum level of groups of Abx-UTI-0.5g-UC-2W, Abx-UTI-1g-UC-2W, Controls, and NS-UC-2W. Only the top 5 most abundant phyla are shown. **(D)** Microbial profile at the genus level of the groups of Abx-UTI-0.5g-UC-2W, Abx-UTI-1g-UC-2W, Control, and NS-UC-2W. Only the top 10 most abundant genera are shown. **(E)** PCoA based on Bray-Curtis distances ASV level showed comparisons of microbial community among groups of Abx-UTI-0.5g-UC-2W, Abx-UTI-1g-UC-2W, Control, and NS-UC-2W. **(F)** Bacterial richness and diversity measured by Chao 1 and Shannon index were calculated at ASV level, and compared among groups of Abx-UTI-0.5g-UC-2W, Abx-UTI-1g-UC-2W, Control, and NS-UC-2W. **(G)** Microbial profile at the phylum level of groups Abx-UTI-0.5g-UC-2W, Abx-UTI-1g-UC-2W, Control, and NS-UC-2W. Only the top 5 most abundant phyla are shown. **(H)** Microbial profile at the genus level of groups of Abx-UTI-0.5g-UC-2W, Abx-UTI-1g-UC-2W, Control, and NS-UC-2W. Only the top 10 most abundant genera are shown.

The composition of the bacterial community in the Abx-UTI-0.5g-UC-2W and Abx-UTI-1g-UC-2W groups was different from that in the control and NS-UC-2W groups (FDR < 0.05) (**Figure 2A**); however, the difference was not significant between the Abx-UTI-0.5g-UC-2W and control groups (R^2 = ^0.091, FDR = 0.520) (**Figure 2A**). Bacterial richness and diversity were not different between the Abx-UTI-0.5g-UC-2W/Abx-UTI-1g-UC-2W and control/NS-UC-2W groups (FDR > 0.05) (**Figure 2B**).

Proteobacteria and Firmicutes were the most abundant bacterial phyla in the control and NS-UC-2W groups (**Figure 2C**). The abundance of Firmicutes was lower in the Abx-UTI-0.5g-UC-2W and Abx-UTI-1g-UC-2W groups than in the control and NS-UC-2W groups (**Figure 2C**). However, no significant differences were found between the Abx-UTI and control/NS-UC-2W groups at the bacterial phylum level (FDR > 0.05). Bacillus was the dominant bacterial genus in the Abx-UTI-0.5g-UC-2W, Abx-UTI-1g-UC-2W and control groups (16.88%, 27.27% and 43.03%, respectively), whereas Streptococcus was the most abundant bacterial genus in the NS-UC-2W group (12.90%) (**Figure 2D**). Although the abundance of several bacterial genera, such as Bacillus, Sphingomonas and Streptococcus, was different between the Abx-UTI and control/NS-UC-2W groups (**Figure 2D**), the differences were not significant (FDR > 0.05).

##### 3.1.2.2 Administration of Abx-UTI via UC for 1 week

Abx-UTI were administered via UC for 1 week, and their effects on the urinary microbiota were examined. The composition of the bacterial community in the Abx-UTI-0.5g-UC-1W group was not different from that in the control and NS-UC-1W groups (FDR > 0.05) (**Figure 2E**); however, the composition of bacterial community in the Abx-UTI-1g-UC-1W group was significantly different from that in the control and NS-UC-1W groups (FDR < 0.05) (**Figure 2E**) while the bacterial diversity did not show difference (**Figure 2F**).

The two most abundant bacterial phyla in the Abx-UTI-0.5g-UC-1W, control and NC-UC-1W groups were Proteobacteria and Firmicutes, whereas the two most abundant phyla in the Abx-UTI-1g-UC-1W group were Firmicutes and Bacteroidota (**Figure 2G**). The most abundant bacterial genus in the four groups was different. For example, Escherichia–Shigella accounted for 11.34% of genera in the Abx-UTI-0.5g-UC-1W group, Bacteroides accounted for 14.74% of genera in the Abx-UTI-1g-UC-1W group, Bacillus accounted for 37.64% of genera in the control group and Proteus accounted for 29.36% of genera in the NS-UC-1W group (**Figure 2H**). When bacterial phyla and genera accounting for >1% of the total bacterial composition were compared between the Abx-UTI and control/NS-UC-1W groups, no significant differences were observed (FDR > 0.05).

#### 3.1.3 Rats administered Abx-UTI via UC for 1 week exhibited distinct bacterial isolates

Urine samples were cultured aerobically and anaerobically for detecting live bacteria. Escherichia coli was the dominant bacterium in 63.64% of samples (7/11 groups) with 10^5^ CFU/mL, Enterococcus faecalis was the dominant bacterium in 45.45% of samples (5/11 groups) with >10^5^ CFU/mL, Proteus mirabilis was the dominant bacterium in 18.18% samples (2/11 groups) and Pseudomonas aeruginosa was the dominant bacterium in 18.18% samples (2/11 groups) with >10^5^ CFU/mL (**Table 1**). However, the abovementioned common bacteria were not isolated from urine samples in the Abx-UTI-1g-UC-1W group. This group had Alcaligenes faecalis with 1 CFU/mL and Staphylococcus cohnii with 4 CFU/mL, which were not present in other groups.

**Table 1:** Bacterial isolates in urine.

Group		Bacteria	Probability scores (%)	CFU/mL
Antibiotics for enteritis	Abx-enteritis-0.5g-G-2W	*Escherichia coli*	100	10^5^
	Abx-enteritis-1g-G-2W	*Enterococcus faecalis*	100	10^5^
Antibiotics for UTI	Abx-UTI-0.5g-G-2W	*Escherichia coli*	100	10^5^
	Abx-UTI-1g-G-2W	*Enterococcus faecalis*	100	10^5^
	Abx-UTI-0.5g-UC-2W	*Escherichia coli*	100	10^5^
	Abx-UTI-1g-UC-2W	*Proteus mirabilis*	100	10^5^
		*Escherichia coli*	100	10^5^
	Abx-UTI-0.5g-UC-1W	*Proteus mirabilis*	100	10^5^
		*Enterococcus faecalis*	100	10^5^
	Abx-UTI-1g-UC-1W	*Alcaligenes faecalis*	99	1
		*Staphylococcus cohnii*	99	4
Sham	NS-UC-1W	*Pseudomonas aeruginosa*	100	10^5^
		*Escherichia coli*	100	10^5^
		*Enterococcus faecalis*	100	10^5^
	NS-UC-2W	*Pseudomonas aeruginosa*	99	10^5^
		*Escherichia coli*	100	10^5^
		*Enterococcus faecalis*	100	10^5^
Control	Control	*Escherichia coli*	100	10^5^

Anti-UTI, anti-urinary tract infection; G, gavage; NS, normal saline; UC, urinary catheterization; W, week.

### 3.2 Safety assessment

#### 3.2.1 Abx-enteritis and Abx-UTI altered the faecal microbiota

Given that antibiotics can damage the faecal microbiota (Blaser, 2016), the effects of Abx-enteritis and Abx-UTI on the faecal microbiota of rats were examined to assess the safety of these antibiotics.

**Figure 3:**
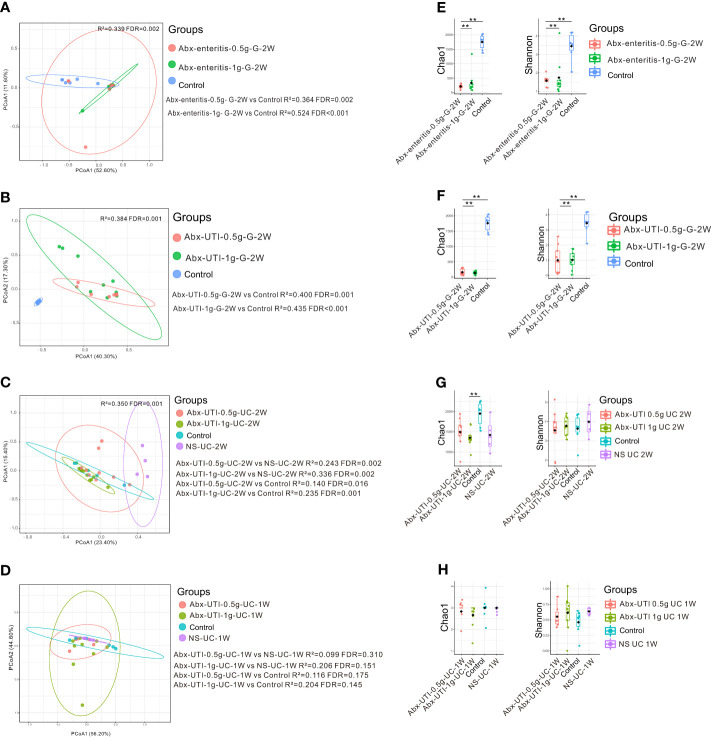
Faecal microbiota alterations in rats. **(A)** PCoA based on Bray-Curtis distances ASV level showed comparisons of microbial community among groups of Abx-UTI-0.5g-G-2W, Abx-UTI-1g-G-2W, and Controls. **(B)** PCoA based on Bray-Curtis distances ASV level showed comparisons of microbial community among groups of Abx-UTI-0.5g-G-2W, Abx-UTI-1g-G-2W, and Controls. **(C)** PCoA based on Bray-Curtis distances ASV level showed comparisons of microbial community among groups of Abx-UTI-0.5g-UC-2W, Abx-UTI-1g-UC-2W, Controls, and NS-UC-2W. **(D)** PCoA based on Bray-Curtis distances ASV level showed comparisons of microbial community among groups of Abx-UTI-0.5g-UC-1W, Abx-UTI-1g-UC-1W, Controls, and NS-UC-1W. **(E)** Bacterial richness and diversity measured by Chao 1 and Shannon index were calculated at ASV level, and compared among groups of Abx-enteritis-0.5g-G-2W, Abx-enteritis-1g-G-2W, and Controls. **(F)** Bacterial richness and diversity measured by Chao 1 and Shannon index were calculated at ASV level, and compared among groups of Abx-UTI-0.5g-G-2W, Abx-UTI-1g-G-2W, and Controls. **(G)** Bacterial richness and diversity measured by Chao 1 and Shannon index were calculated at ASV level, and compared among groups of Abx-UTI-0.5g-UC-2W, Abx-UTI-1g-UC-2W, Controls, and NS-UC-2W. **(H)** Bacterial richness and diversity measured by Chao 1 and Shannon index were calculated at ASV level, and compared among groups of Abx-UTI-0.5g-UC-1W, Abx-UTI-1g-UC-1W, Controls, and NS-UC-1W. ** represented FDR<0.01.

Compared with rats in the control group, those administered Abx-enteritis and Abx-UTI via gavage had different faecal microbiota, irrespective of the doses of the antibiotics (FDR < 0.05) (**Figures 3A, B**). However, the composition of bacterial community in the Abx-UTI-0.5g-UC-2W and Abx-UTI-1g-UC-2W groups was different from that in the control and NS-UC-2W groups (FDR < 0.05) (**Figure 3C**). These findings were not observed after 1 week of drug administration (FDR > 0.05; **Figure 3D**). Furthermore, bacterial richness (Chao 1 index) and bacterial diversity (Shannon index) were lower in the two Abx-enteritis groups than in the control group (FDR < 0.05) (**Figures 3E, F**). Bacterial richness was lower in the Abx-UTI-1g-UC-2W group than in the control group (FDR < 0.05) (**Figure 3G**); however, it was not different between the Abx-UTI-1g-UC-1W and control/NS-UC-1W groups (FDR > 0.05) (**Figure 3H**). Additionally, the number of bacterial phyla and genera showing different from controls/sham group was higher in the faecal microbiota of rats administered Abx-enteritis via gavage or Abx-UTI via UC for 2 weeks than in that of rats administered Abx-UTI via UC for 1 week (**Figures S1A–D**).

#### 3.2.2 Antibiotics played a role in body weight, food and water intake and faeces and urine output

**Figure 4:**
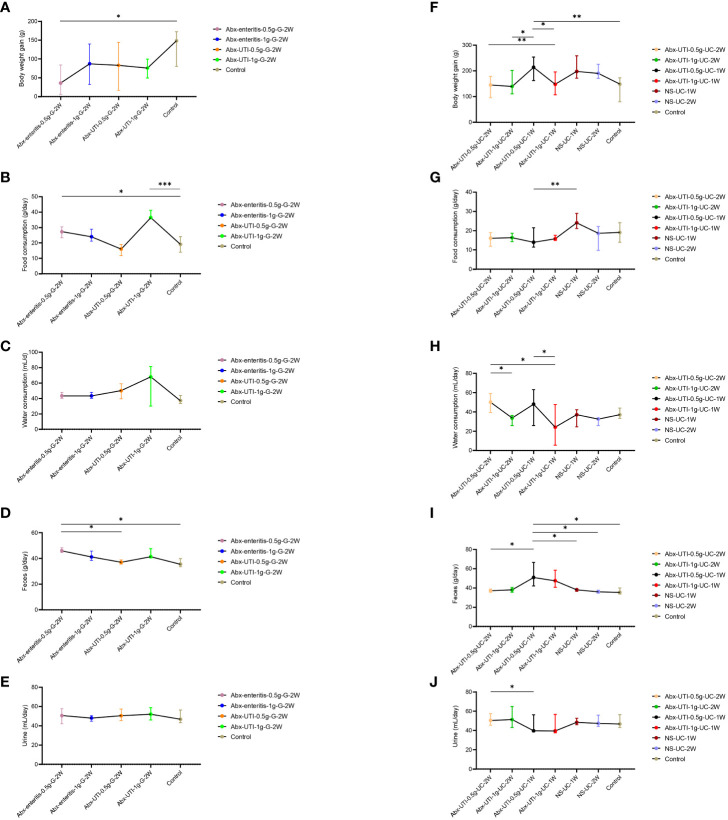
Comparisons of body weight, food and water consumption, faeces and urine output among groups. **(A)** Comparisons of body weight gain among groups of Abx-enteritis-0.5g-G-2W, Abx-enteritis-1g-G-2W, Abx-UTI-0.5g-G-2W, Abx-UTI-1g-G-2W, and Controls. **(B)** Comparisons of food consumption among groups of Abx-enteritis-0.5g-G-2W, Abx-enteritis-1g-G-2W, Abx-UTI-0.5g-G-2W, Abx-UTI-1g-G-2W, and Controls. **(C)** Comparisons of water consumption among groups of Abx-enteritis-0.5g-G-2W, Abx-enteritis-1g-G-2W, Abx-UTI-0.5g-G-2W, Abx-UTI-1g-G-2W, and Controls. **(D)** Comparisons of feces output among groups of Abx-enteritis-0.5g-G-2W, Abx-enteritis-1g-G-2W, Abx-UTI-0.5g-G-2W, Abx-UTI-1g-G-2W, and Controls. **(E)** Comparisons of urine output among groups of Abx-enteritis-0.5g-G-2W, Abx-enteritis-1g-G-2W, Abx-UTI-0.5g-G-2W, Abx-UTI-1g-G-2W, and Controls. **(F)** Comparisons of body weight gain among groups of Abx-UTI-0.5g-UC-2W, Abx-UTI-1g-UC-2W, Abx-UTI-0.5g-UC-1W, Abx-UTI-1g-UC-1W, NS-UC-1W, NS-UC-2W, and Controls. **(G)** Comparisons of food consumption gain among groups of Abx-UTI-0.5g-UC-2W, Abx-UTI-1g-UC-2W, Abx-UTI-0.5g-UC-1W, Abx-UTI-1g-UC-1W, NS-UC-1W, NS-UC-2W, and Controls. **(H)** Comparisons of water consumption gain among groups of Abx-UTI-0.5g-UC-2W, Abx-UTI-1g-UC-2W, Abx-UTI-0.5g-UC-1W, Abx-UTI-1g-UC-1W, NS-UC-1W, NS-UC-2W, and Controls. **(I)** Comparisons of feces output among groups of Abx-UTI-0.5g-UC-2W, Abx-UTI-1g-UC-2W, Abx-UTI-0.5g-UC-1W, Abx-UTI-1g-UC-1W, NS-UC-1W, NS-UC-2W, and Controls. **(J)** Comparisons of urine output among groups of Abx-UTI-0.5g-UC-2W, Abx-UTI-1g-UC-2W, Abx-UTI-0.5g-UC-1W, Abx-UTI-1g-UC-1W, NS-UC-1W, NS-UC-2W, and Controls. *, **, ***represented FDR<0.05, FDR<0.01 and FDR<0.001, respectively.

The body weight, food and water intake and faeces and urine output of rats (pre-and post-treatment) using metabolic cages. Body weight gain was lower in the Abx-enteritis-0.5g-G-2W group than in the control group. However, food intake and faeces output were higher in the Abx-enteritis-0.5g-G-2W group than in the control group (P < 0.05) (**Figures 4A, B, D**). There were no difference in water consumption and urine production among each group after gavage (**Figures 4C, E**). Meanwhile, the body weight gain and faeces output were higher in the Abx-UTI-0.5-UC-1W group than in the control group (P < 0.05) (**Figure 4F**). Whereas the food consumption, water consumption, feces output and urine production were no changes between the urinary catheterisation group and the control group (**Figures 4G–J**).

#### 3.2.3 Antibiotics did not alter the structure and function of the bladder and kidney

**Figure 5:**
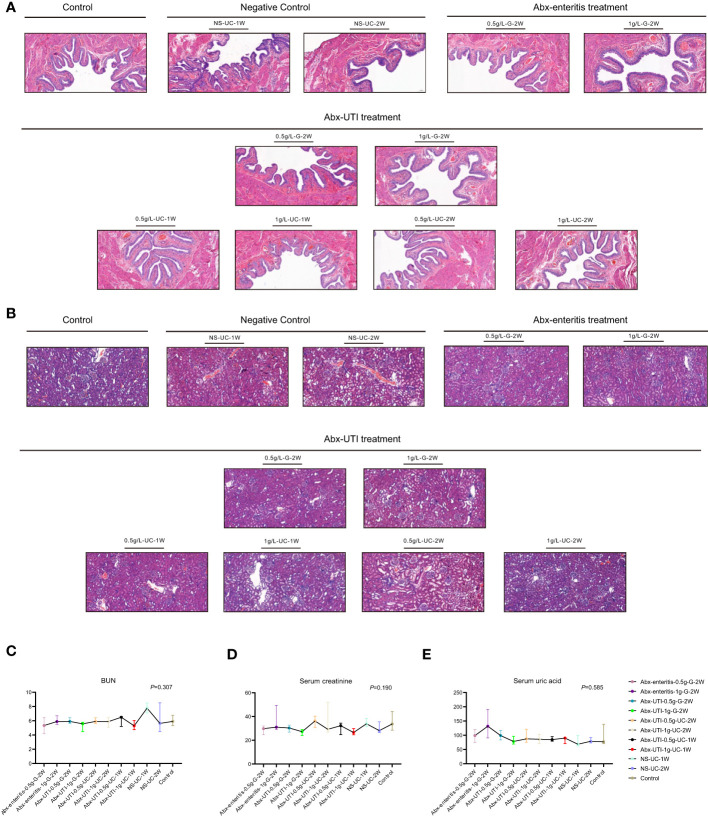
Structure of bladder and kidney, kidney functions. **(A)** Structure of bladder. H&E staining was performed; **(B)** Structure of kidney. H&E staining was performed; **(C)** Comparisons of kidney function. The kidney function comparisons were detected by **(C)** BUN; **(D)** Serum creatinine and **(E)** Serum uric acid.

To determine the effects of Abx-enteritis and Abx-UTI on the bladder, structural changes were observed in rat bladder tissues. H&E staining revealed that the mucosal and muscular layers of the bladder tissue were intact and inflammatory cell infiltration was not observed in the control group (**Figure 5A**). Additionally, the transitional epithelial cell layer was closely arranged, and exfoliated epithelial cells were not observed. The histological features of the Abx-enteritis and Abx-UTI groups were not significantly different from those of the control and sham groups.

Given that nephrotoxicity is a common adverse effect of antibiotics, assessing the structure and function of the kidney is necessary. In the control group, the glomerular structure was intact and well demarcated, and the capillary loops were unobstructed and intact. Additionally, the renal tubular epithelial cells were closely arranged without shedding or necrosis (**Figure 5B**). Glomerular mesangial cell hyperplasia and inflammatory cell infiltration were observed in all antibiotic-treated groups. However, no significant differences were observed between the antibiotic-treated and control/sham groups (**Figure 5B**).

The levels of blood urine nitrites, serum creatinine and serum uric acid were evaluated to assess kidney function. As shown in **Figures 5C–E**, the levels of these indicators were not significantly different between the Abx-enteritis/Abx-UTI and control/sham groups, irrespective of the administration dose and frequency (P > 0.05).

## 4 Discussion

In this study, the effects of Abx-enteritis and Abx-UTI on the urinary microbiota were compared via 16S rRNA sequencing and culture. In addition, the safety profile of these antibiotics was assessed by evaluating the body weight, bladder and kidney structure and kidney function of rats.

Administration of Abx-enteritis and Abx-UTI via gavage did not alter the urinary microbiota in rats; however, administration of Abx-UTI at a dose of 1 g/L via UC for either 1 week or 2 weeks altered the bacterial community in their urine. Administration of Abx-enteritis via gavage altered the faecal bacterial community in rats, whereas administration of Abx-UTI via UC for 1 week did not alter the faecal microbial community. In addition, the rats treated with Abx-UTI via UC for 1 week had the least number of bacterial phyla and genera showing different from the group of controls and sham in the faeces. Therefore, administration of antibiotics at a dose of 1 g/L via UC for 1 week may be more suitable for disrupting the urinary microbiota without affecting the faecal microbiota (Velmurugan, 2018; Ampatzoglou et al., 2022).

Rats administered Abx-enteritis and Abx-UTI via gavage and UC had slightly increased bacterial diversity in the urinary microbiota, whereas rats administered Abx-enteritis and Abx-UTI via gavage had substantially decreased bacterial diversity in the faecal microbiota. Bacterial diversity in the faecal microbiota of rats administered Abx-UTI via UC was similar to that of rats in the control and sham groups. Bacterial diversity in human urine tends to increase in unhealthy states, such as urinary incontinence (Shoskes et al., 2016), an overactive bladder (Wu et al., 2017), and interstitial cystitis (Siddiqui et al., 2012). However, bacterial diversity in human faeces tends to decrease in unhealthy states, including psoriatic arthritis (Scher et al., 2015), and systemic lupus erythematosus (Liu et al., 2021). Therefore, from the perspective of an unhealthy state induced by antibiotic-associated gut dysbiosis (Thänert et al., 2022), the UC route may be better than the gavage route for analyzing the urinary microbiota of animals (as evidenced by the changes observed in the Abx-UTI-1g-UC-1W group).

We noticed that bacterial isolates were less in the Abx-UTI-1g-UC-1W group than in other groups, indicating that the administration of Abx-UTI via UC for 1 week can decrease the proportion of living microbes in the bladder of rats.

Administration of Abx-enteritis via gavage induced a decrease in weight gain with an increase in food consumption, which is consistent with the results of a previous study (Bongers et al., 2022). Bongers et al. reported that mice lost weight after they were administered a cocktail of ampicillin, neomycin, vancomycin and metronidazole (Bongers et al., 2022). Additionally, Xu et al. (2021) reported that food consumption was increased among mice administered Abx-enteritis via gavage instead of via drinking water for 1 week because appetite was not affected in the gavage route (Xu et al., 2021). Similarly, in our pre-experimental study, we found that almost all rats administered antibiotics via drinking water died at the end of week 2 because their water intake was reduced owing to the strong smell of the drinking water. Notably, rats administered antibiotics via UC did not lose weight, and the intake of food and water was not altered. Therefore, when the growth of rats is considered while investigating the urinary microbiota, the UC route may be a safe option.

Administration of Abx-enteritis and Abx-UTI did not affect the bladder and kidney tissues and kidney function of rats. However, Xu et al. reported that mice administered antibiotics via gavage had increased serum creatinine levels (Xu et al., 2021). This discrepancy may be attributed to the use of mice instead of rats in their study. Based on the findings of this study, we conclude that administration of either Abx-enteritis or Abx-UTI via gavage or UC for <2 weeks is safe for the bladder and kidney of rats.

A limitation of this study is that we did not collect urine samples at intervals because we speculated that frequent UC might lead to urinary tissue damage in rats.

## 5 Conclusions

Based on 16S rRNA sequencing, urine culture and the safety profiles, administration of Abx-UTI at a dose of 1g for 1 week may be an appropriate option for antibiotic pre-treatment to examine the urinary microbiota of rats.

## Data availability statement

The datasets presented in this study can be found in online repositories. The names of the repository/repositories and accession number(s) can be found in the article/**Supplementary Material**.

## Ethics statement

The present study was approved by the animal care committee of Jiangnan University [ref. JN.No20201230S1200430(372)].

## Author contributions

Conceptualization: FL, WG, YG, QX and NF. Methodology: LH, JS, YT, YS, PJ, SW, JH, HL, ZX. Software: FL. Validation: WG, YG, NF. Formal analysis: FL. Investigation: LH. Resources: NF. Data curation: FL, LH, WG. Writing—original draft preparation: FL, WG. Writing—review and editing: FL, WG. Visualization: NF. Supervision: NF. Project administration: LH, JS. Funding acquisition: NF. All authors have read and agreed to the published version of the manuscript. All authors contributed to the article and approved the submitted version.
