# Supplementary material for: Can antibiotics for enteritis or for urinary tract infection disrupt the urinary microbiota in rats?
Source: Front Cell Infect Microbiol. 2023 Jun 28;13:1169909. doi: 10.3389/fcimb.2023.1169909 (PMC10338079; doi:10.3389/fcimb.2023.1169909)
Supplement: Supplementary file 1 [file DataSheet_1.zip › Data Sheet 1/Related Article 1/Figure 2 Abx-UTI on UM via UC.pdf]

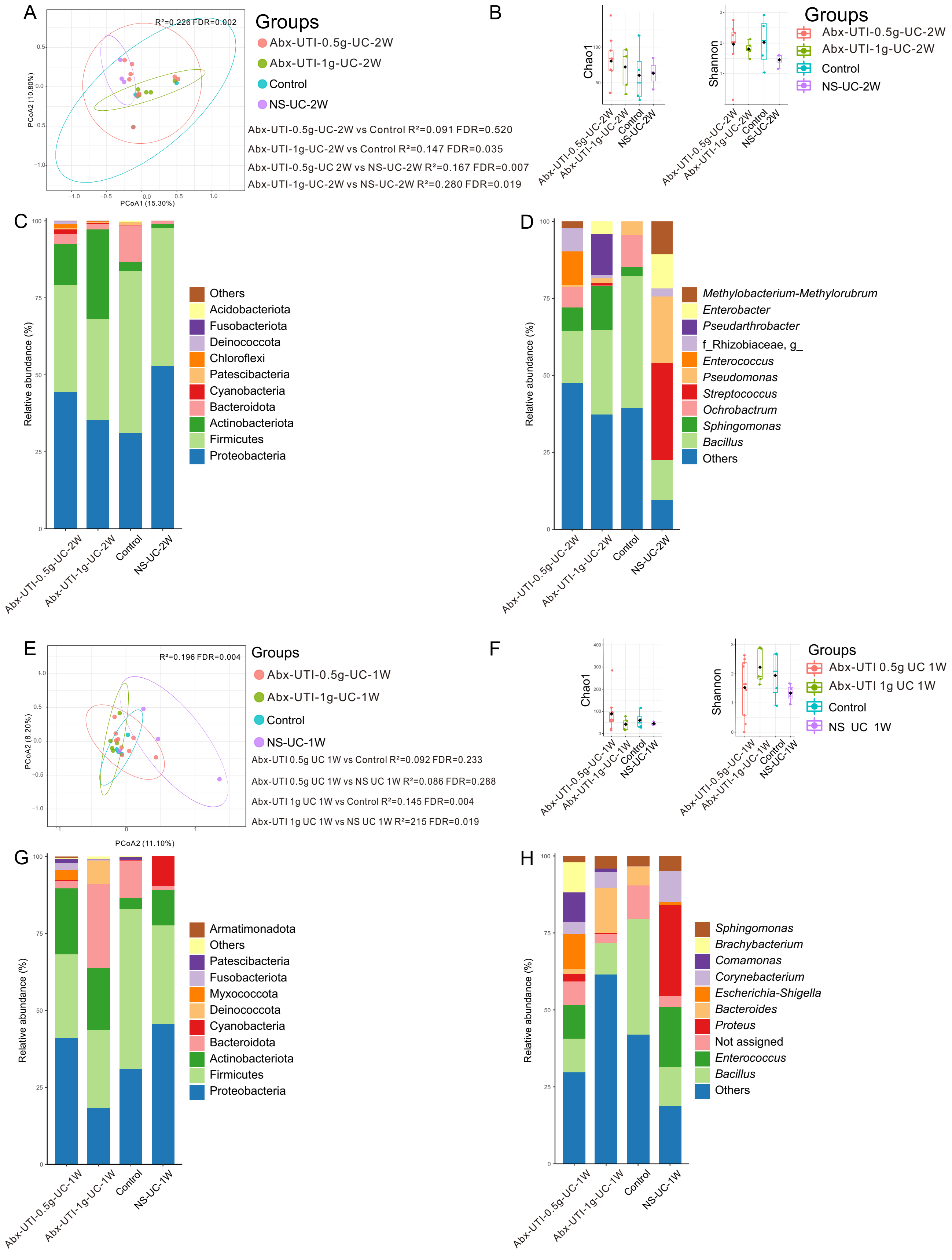
A
R²=0.226 FDR=0.002
PCoA1 (15.30%)
PCoA2 (10.80%)
Groups
Abx-UTI-0.5g-UC-2W
Abx-UTI-1g-UC-2W
Control
NS-UC-2W
Abx-UTI-0.5g-UC-2W vs Control R²=0.091 FDR=0.520
Abx-UTI-1g-UC-2W vs Control R²=0.147 FDR=0.035
Abx-UTI-0.5g-UC 2W vs NS-UC-2W R²=0.167 FDR=0.007
Abx-UTI-1g-UC-2W vs NS-UC-2W R²=0.280 FDR=0.019
B
Chao1
Shannon
Groups
Abx-UTI-0.5g-UC-2W
Abx-UTI-1g-UC-2W
Control
NS-UC-2W
C
Relative abundance (%)
Others
Acidobacteriota
Fusobacteriota
Deinococcota
Chloroflexi
Patescibacteria
Cyanobacteria
Bacteroidota
Actinobacteriota
Firmicutes
Proteobacteria
Abx-UTI-0.5g-UC-2W
Abx-UTI-1g-UC-2W
Control
NS-UC-2W
D
Relative abundance (%)
Methylobacterium-Methylorubrum
Enterobacter
Pseudarthrobacter
f_Rhizobiaceae, g_
Enterococcus
Pseudomonas
Streptococcus
Ochrobactrum
Sphingomonas
Bacillus
Others
E
R²=0.196 FDR=0.004
PCoA2 (11.10%)
PCoA2 (8.20%)
Groups
Abx-UTI-0.5g-UC-1W
Abx-UTI-1g-UC-1W
Control
NS-UC-1W
Abx-UTI 0.5g UC 1W vs Control R²=0.092 FDR=0.233
Abx-UTI 0.5g UC 1W vs NS UC 1W R²=0.086 FDR=0.288
Abx-UTI 1g UC 1W vs Control R²=0.145 FDR=0.004
Abx-UTI 1g UC 1W vs NS UC 1W R²=215 FDR=0.019
F
Chao1
Shannon
Groups
Abx-UTI 0.5g UC 1W
Abx-UTI 1g UC 1W
Control
NS UC 1W
G
Relative abundance (%)
Armatimonadota
Others
Patescibacteria
Fusobacteriota
Myxococcota
Deinococcota
Cyanobacteria
Bacteroidota
Actinobacteriota
Firmicutes
Proteobacteria
Abx-UTI-0.5g-UC-1W
Abx-UTI-1g-UC-1W
Control
NS-UC-1W
H
Relative abundance (%)
Sphingomonas
Brachybacterium
Comamonas
Corynebacterium
Escherichia-Shigella
Bacteroides
Proteus
Not assigned
Enterococcus
Bacillus
Others
