# Supplementary material for: Can antibiotics for enteritis or for urinary tract infection disrupt the urinary microbiota in rats?
Source: Front Cell Infect Microbiol. 2023 Jun 28;13:1169909. doi: 10.3389/fcimb.2023.1169909 (PMC10338079; doi:10.3389/fcimb.2023.1169909)
Supplement: Supplementary file 1 [file DataSheet_1.zip › Data Sheet 1/Related Article 1/Figure 3 Antibiotics on fecal microbiome.pdf]

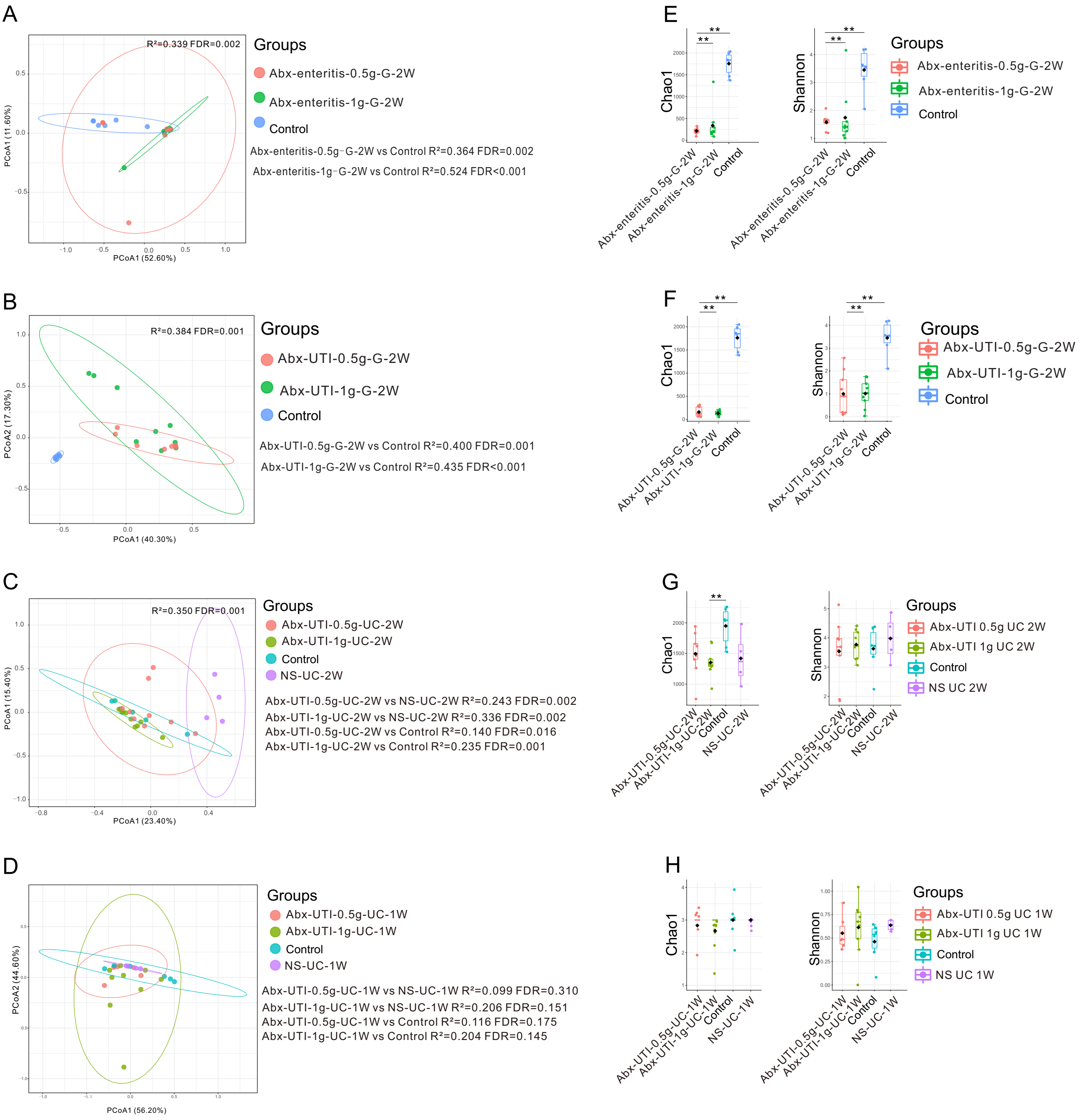
A
R²=0.339 FDR=0.002
Groups
Abx-enteritis-0.5g-G-2W
Abx-enteritis-1g-G-2W
Control
Abx-enteritis-0.5g-G-2W vs Control R²=0.364 FDR=0.002
Abx-enteritis-1g-G-2W vs Control R²=0.524 FDR<0.001
PCoA1 (52.60%)
PCoA1 (11.60%)
B
R²=0.384 FDR=0.001
Groups
Abx-UTI-0.5g-G-2W
Abx-UTI-1g-G-2W
Control
Abx-UTI-0.5g-G-2W vs Control R²=0.400 FDR=0.001
Abx-UTI-1g-G-2W vs Control R²=0.435 FDR<0.001
PCoA1 (40.30%)
PCoA2 (17.30%)
C
R²=0.350 FDR=0.001
Groups
Abx-UTI-0.5g-UC-2W
Abx-UTI-1g-UC-2W
Control
NS-UC-2W
Abx-UTI-0.5g-UC-2W vs NS-UC-2W R²=0.243 FDR=0.002
Abx-UTI-1g-UC-2W vs NS-UC-2W R²=0.336 FDR=0.002
Abx-UTI-0.5g-UC-2W vs Control R²=0.140 FDR=0.016
Abx-UTI-1g-UC-2W vs Control R²=0.235 FDR=0.001
PCoA1 (23.40%)
PCoA1 (15.40%)
D
Groups
Abx-UTI-0.5g-UC-1W
Abx-UTI-1g-UC-1W
Control
NS-UC-1W
Abx-UTI-0.5g-UC-1W vs NS-UC-1W R²=0.099 FDR=0.310
Abx-UTI-1g-UC-1W vs NS-UC-1W R²=0.206 FDR=0.151
Abx-UTI-0.5g-UC-1W vs Control R²=0.116 FDR=0.175
Abx-UTI-1g-UC-1W vs Control R²=0.204 FDR=0.145
PCoA1 (56.20%)
PCoA2 (44.60%)
E
Chao1
Shannon
Groups
Abx-enteritis-0.5g-G-2W
Abx-enteritis-1g-G-2W
Control
F
Chao1
Shannon
Groups
Abx-UTI-0.5g-G-2W
Abx-UTI-1g-G-2W
Control
G
Chao1
Shannon
Groups
Abx-UTI 0.5g UC 2W
Abx-UTI 1g UC 2W
Control
NS UC 2W
H
Chao1
Shannon
Groups
Abx-UTI 0.5g UC 1W
Abx-UTI 1g UC 1W
Control
NS UC 1W
