# Supplementary material for: Can antibiotics for enteritis or for urinary tract infection disrupt the urinary microbiota in rats?
Source: Front Cell Infect Microbiol. 2023 Jun 28;13:1169909. doi: 10.3389/fcimb.2023.1169909 (PMC10338079; doi:10.3389/fcimb.2023.1169909)
Supplement: Supplementary file 1 [file DataSheet_1.zip › Data Sheet 1/Related Article 1/Supplementary Material.DOCX]

Supplementary Material

Can antibiotics for enteritis or for urinary tract infection disrupt the urinary microbiota in rats？

Fengping Liu ^1^†, Lei Hu^2^†, Jiayi Sheng^2^†, Yifan Tang^2^, Yifan Sun^2^, Peng Jiang^2^, Shichao Wei^2^, Jialin Hu^2^, Hao Lin^2^, Zhenyi Xu^2^, Wei Guo^2^*, Yifeng Gu^3^*, and Ninghan Feng^2^*

*** Correspondence:**

Wei Guo, Yifeng Gu, and Ninghan Feng

WG: 15851852826@163.com; YfG: guyifeng@zju.edu.cn; NhF: [n.feng@njmu.edu.cn](mailto:n.feng@njmu.edu.cn)

# Supplementary Data

Supplements data includes Supplementary File S1：Methods and Figure S1: Numbers of altered taxonomy.

# Supplementary File and Figures

## Supplementary Figures 1


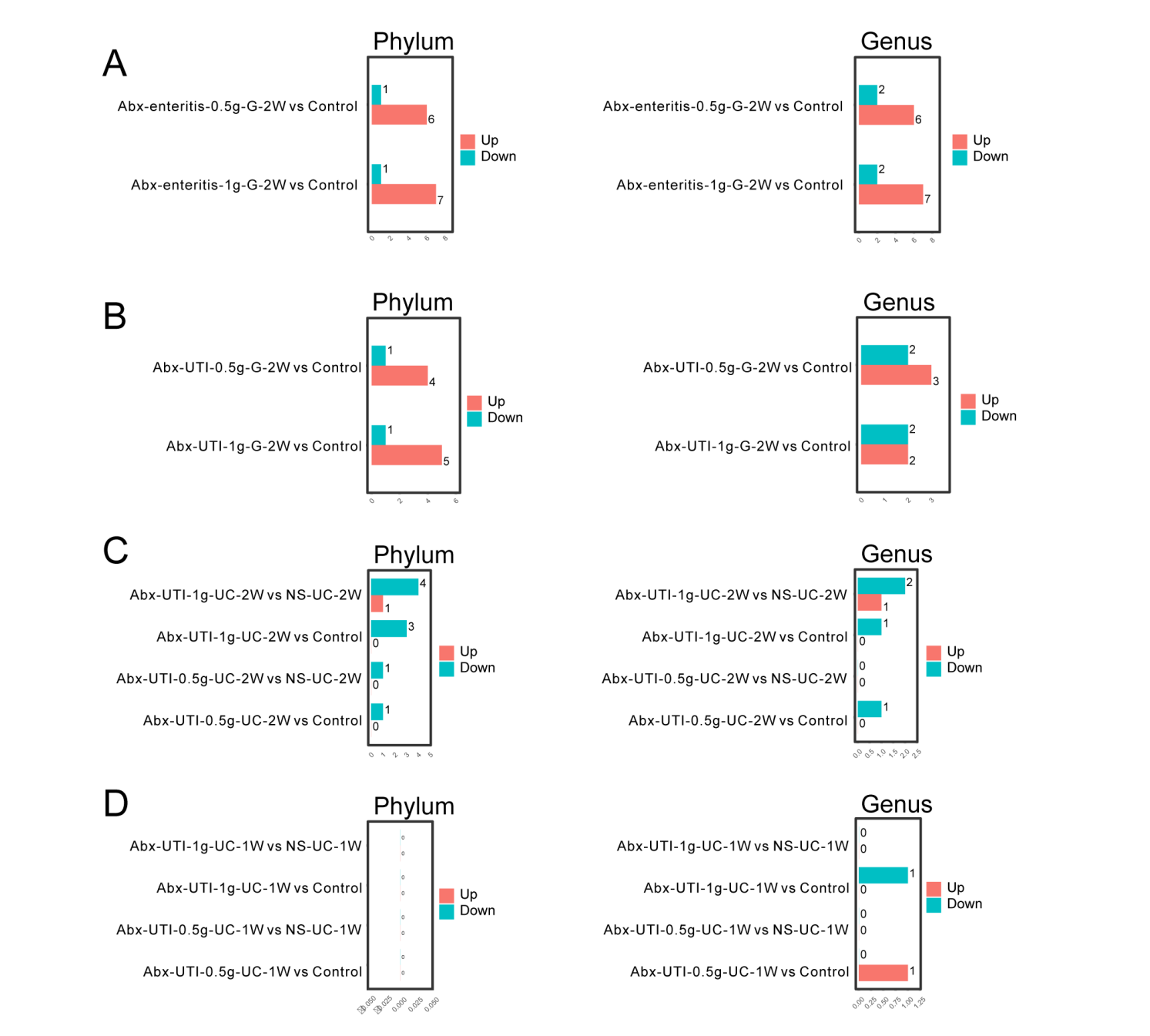


**Supplementary Figure 1.** **Numbers of bacterial phylum and genus differed among groups.**

**(A)** Numbers of bacterial phylum and genus differed between Abx-enteritis-0.5g-G-2W and Control, and between Abx-enteritis-1g-G-2W and Control.

**(B)** Numbers of bacterial phylum and genus differed between Abx-UTI-0.5g-G-2W and Control, and between Abx-UTI-1g-G-2W and Control.

**(C)** Numbers of bacterial phylum and genus differed between Abx-UTI-1g-UC-2W and NS-UC-2W, between Abx-UTI-1g-UC-2W and Control, between Abx-UTI-0.5g-UC-2W and NS-UC-2W, and between Abx-UTI-0.5g-UC-2W and Control.

**(D)** Numbers of bacterial phylum and genus differed between Abx-UTI-1g-UC-1W and NS-UC-1W, between Abx-UTI-1g-UC-1W vs and Control, between Abx-UTI-0.5g-UC-1W and NS-UC-1W, and between Abx-UTI-0.5g-UC-1W and Control.

## Supplementary File 1

**Methods**

**Collection and storage of urine**

Urine samples were collected via UC. Before catheter insertion, the rats were treated with 2% [iodine tincture](javascript:;) to disinfect the abdominal, genital and perineal areas twice. At least 820 μL of urine was obtained and divided into two portions (i.e. 800 μL and 20 μL), which were used for 16S rRNA gene sequencing and urine culture, respectively. All samples were processed in a biosafety cabinet. Samples for 16S rRNA gene sequencing and culture were placed in sterile, DNA- and enzyme-free centrifuge tubes. Samples for 16S rRNA gene sequencing were stored at –80°C within 15 mins of collection. Samples for culture were placed in an insulated sterile container, immediately cooled on ice and transferred to the research personnel within 1 hour. Faecal samples for sequencing were collected after urine was collected and were stored at –80°C until bacterial DNA extraction.

**Bacteria DNA isolation**

Bacterial DNA isolation from urine was described previously[1]. The quantity and quality of the extracted DNA was measured using a NanoDrop ND-1000 spectrophotometer (Thermo Fisher Scientific, Waltham, MA, USA) and agarose gel electrophoresis. Using the isolated DNA as the template, the bacterial 16S rRNA V3-V4 region was PCR-amplified for 32 cycles using universal primers 338F and 806R. The resultant PCR amplicons were purified with Agencourt AMPure XP Beads (Beckman Coulter, Indianapolis, IN, USA) and quantified using the PicoGreen dsDNA Assay Kit (Invitrogen, Carlsbad, CA, USA). Amplicon pools were prepared for sequencing, and the size and quantity of the amplicon library were assessed using an Agilent 2100 Bioanalyzer (Agilent, Santa Clara, MA, USA) and Library Quantification Kit for Illumina (Kapa Biosciences, Woburn, MA, USA), respectively. The libraries were sequenced using the NovaSeq 6000 platform.

**Bioinformatic analysis**

Raw reads of the 16S rRNA gene sequences were trimmed using Cutadapt (cutadapt.readthedocs.io) to remove barcodes and adaptors. The overlapping paired-end reads were merged into a longer tag using FLASH (v1.2.8). Reads were quality trimmed using fdtrim (v0.94) from the 3’ end to remove bases with low-quality scores. Reads shorter than 100bp, with more than 5% Ns, or with an average quality below 20 were discarded. Chimeras were removed using Vsearch (v2.3.4). QIIME2 was used to process the clean reads to generate an ASV table, and taxonomy of microbes was identified using the Silva database (v138). Environmental contaminants of urine samples were removed as previously described[1]. Bacterial ASVs whose counts did not exceed ten times the maximum number of counts in the negative controls were considered as contaminates and removed.

**Bacterial culture and identification in urine.**

10μL of urine was plated on BAP and 10μL on CDC anaerobe 5% sheep blood agars for isolation and incubated aerobically and anaerobically at 35°C for 48h, respectively. Each morphologically distinct colony type was isolated on a different plate of the same media to prepare a pure culture that was used for identification.

We used Matrix-Assisted Laser Desorption Ionization-Time-Of-Flight Mass Spectrometry (MALDI-TOF MS). Briefly, a small portion of a single colony was overlaid with 1μL of a saturated solution of alpha-cyano-4-hydroxycinnamic acid matrix in 50% acetonitrile and 2.5% trifluoroacetic acid (VITEK MSCHCA, bioMérieux), then air dried. Escherichia coli (ATCC 8739) was used for system calibration. Mass spectra were acquired using a VITEK MS Plus (bioMérieux, Marcy l’Etoile) and the bioMérieux VITEK MS (IVD Knowledgebase v.3.2) used to analyze a composite mass spectrum for accurate identification. A probability score between 60% and 99.9% was considered to be a high discrimination value and thus a reliable identification. A probability score <60% was considered to be a low discrimination identification. No identification was resulted when either no match was found for the composite spectra, or the analysis did not obtain enough spectral peaks. Isolates that yielded no identification results were redeposited on the target plate and reanalyzed.

**References**

1. Liu F, Du J, Zhai Q, Hu J, Miller AW, Ren T *et al*. The Bladder Microbiome, Metabolome, Cytokines, and Phenotypes in Patients with Systemic Lupus Erythematosus. *Microbiol Spectr* (2022): e21222.
