# Supplementary figures and images for: Can antibiotics for enteritis or for urinary tract infection disrupt the urinary microbiota in rats?
Source: Front Cell Infect Microbiol. 2023 Jun 28;13:1169909. doi: 10.3389/fcimb.2023.1169909 (PMC10338079; doi:10.3389/fcimb.2023.1169909)

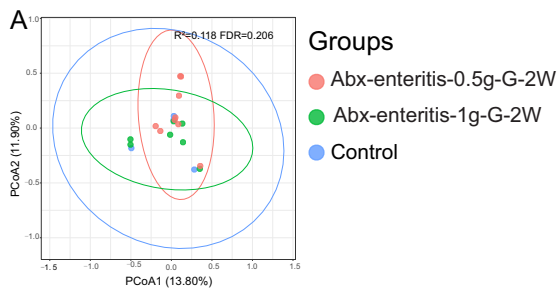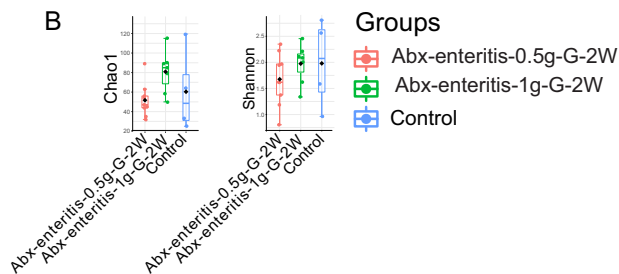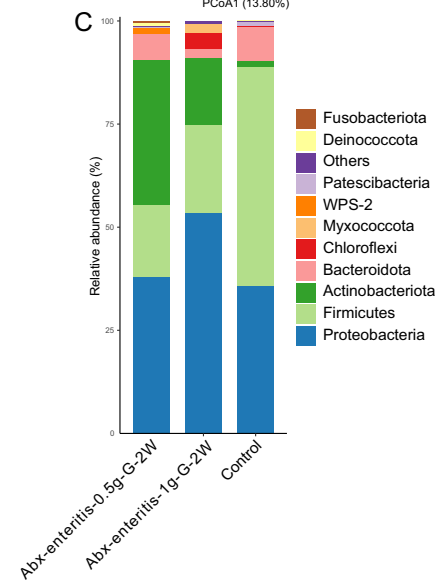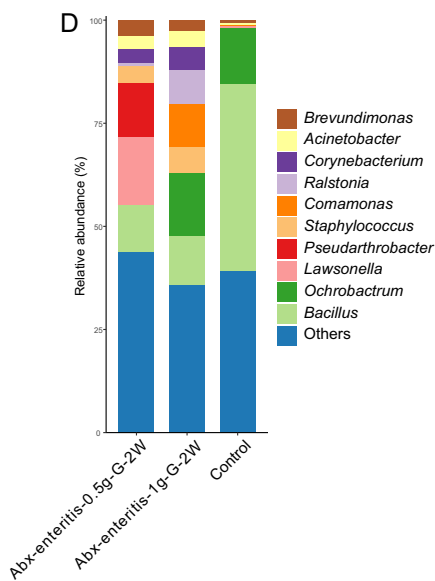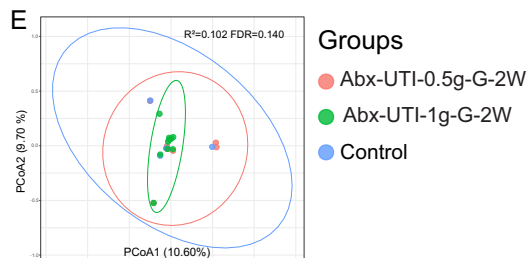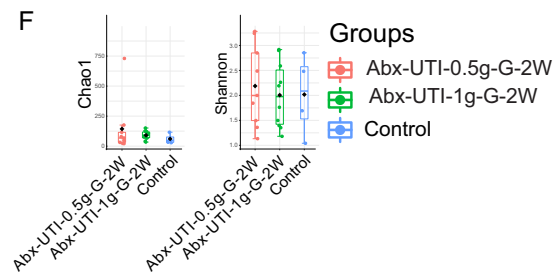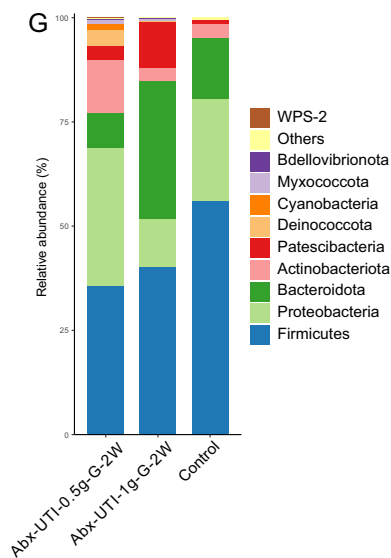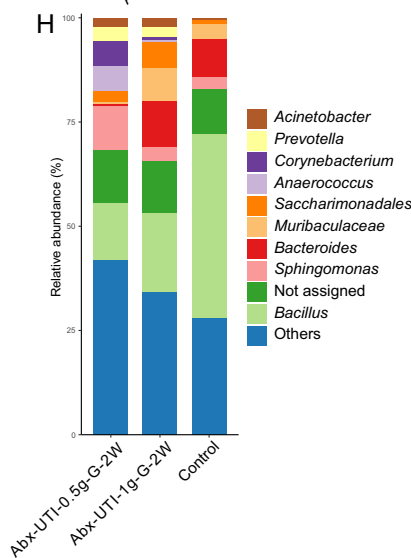

Supplement: Supplementary file 1 [file DataSheet_1.zip › Data Sheet 1/Related Article 1/Figure 1 Abx on UM via Gavage.pdf]

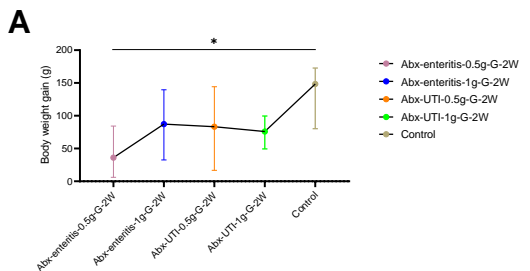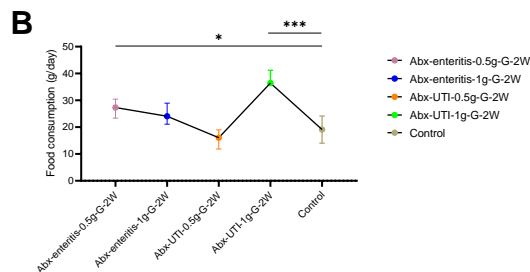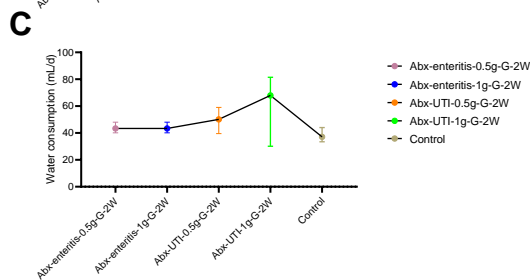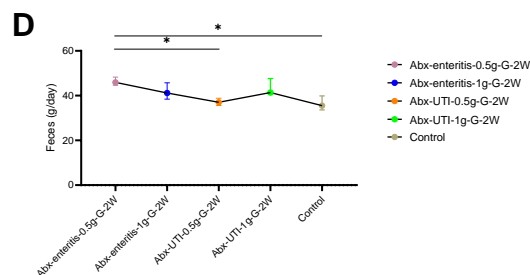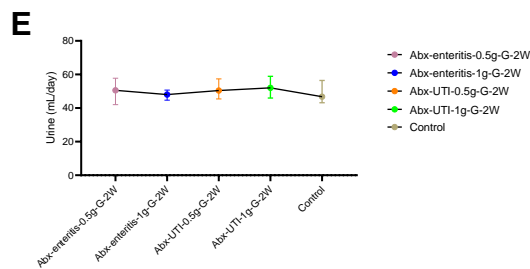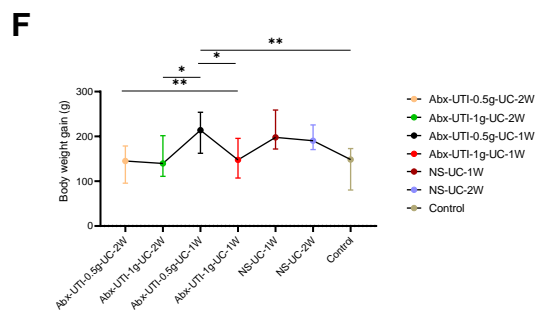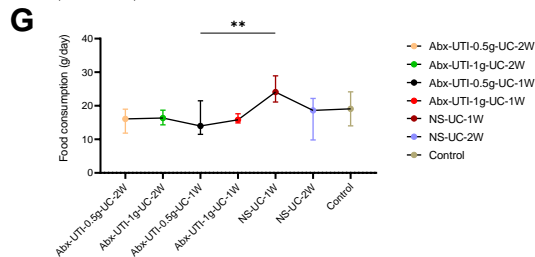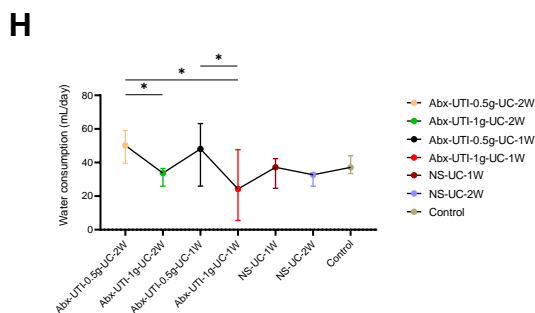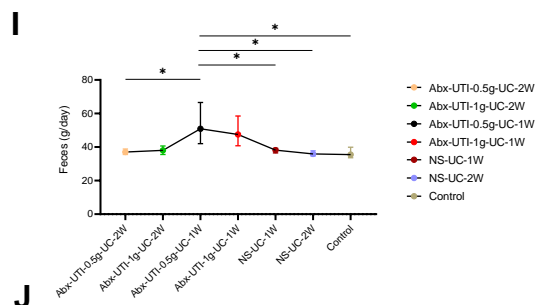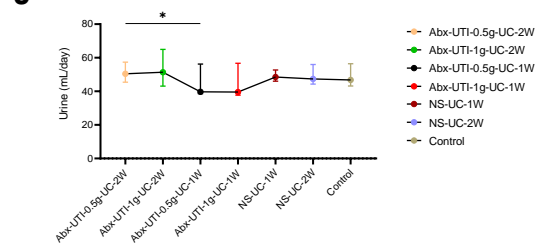

Supplement: Supplementary file 1 [file DataSheet_1.zip › Data Sheet 1/Related Article 1/Figure 4 physical alteration.pdf]

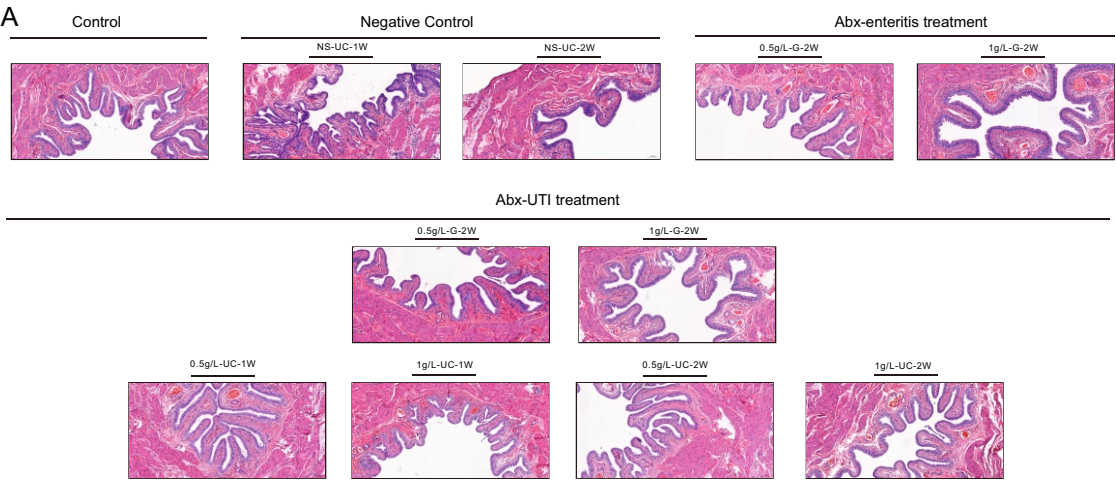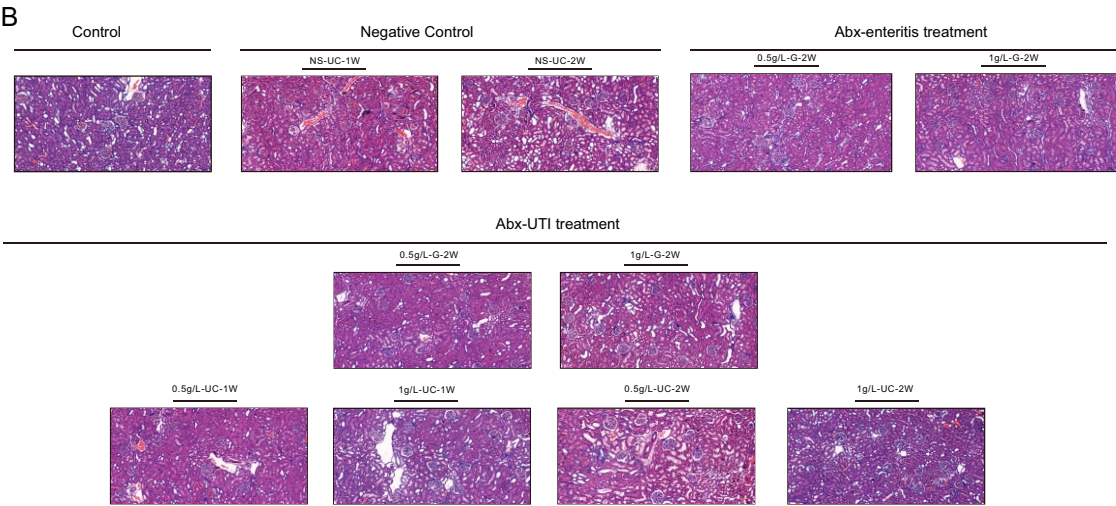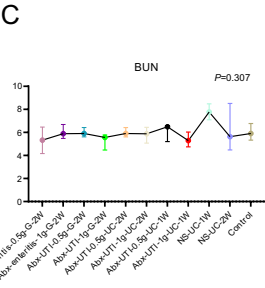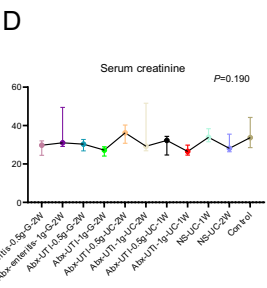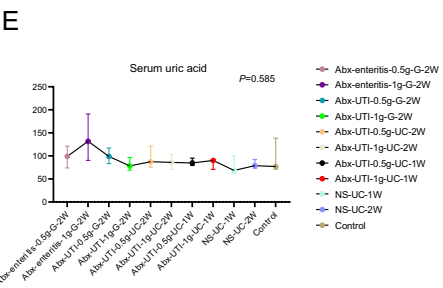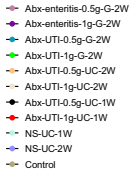

Supplement: Supplementary file 1 [file DataSheet_1.zip › Data Sheet 1/Related Article 1/Figure 5 kidney consturctrue and function.pdf]

A

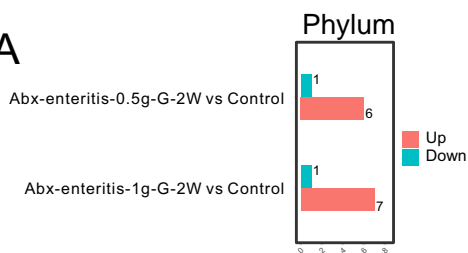

Genus

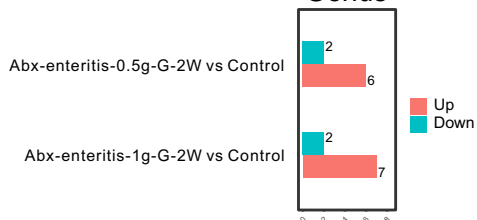

B

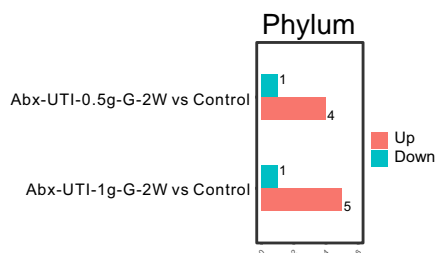

Genus

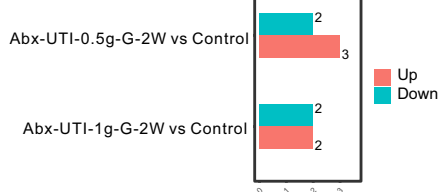

C

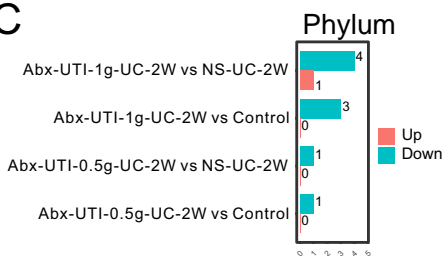

Genus

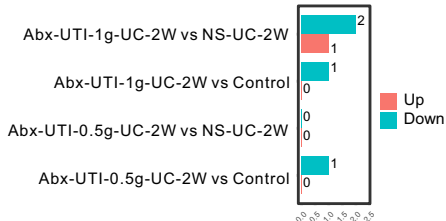

D

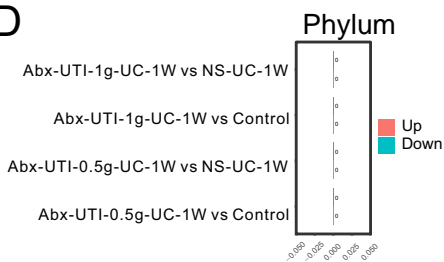

Genus

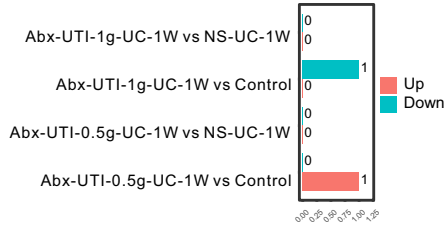

Supplement: Supplementary file 1 [file DataSheet_1.zip › Data Sheet 1/Related Article 1/Figure S1 Numbers of altered taxon.pdf]
